# Frequency of Intimate Partner Violence among an Urban Emergency Department Sample: A Multilevel Analysis

**DOI:** 10.3390/ijerph18010222

**Published:** 2020-12-30

**Authors:** Carol B. Cunradi, William R. Ponicki, Raul Caetano, Harrison J. Alter

**Affiliations:** 1 Prevention Research Center, Pacific Institute for Research and Evaluation, 2150 Shattuck Avenue, Suite 601, Berkeley, CA 94704, USA; bponicki@prev.org (W.R.P.); raul.caetano@utsouthwestern.edu (R.C.); 2 Andrew Levitt Center for Social Emergency Medicine, Berkeley, CA 94703, USA; harrison_alter@levittcenter.org

**Keywords:** intimate partner violence, emergency department, social disadvantage, gender

## Abstract

Intimate partner violence (IPV) is a pervasive public health problem. Within the U.S., urban emergency department (ED) patients have elevated prevalence of IPV, substance use, and other social problems compared to those in the general household population. Using a social-ecological framework, this cross-sectional study analyzes the extent to which individual, household, and neighborhood factors are associated with the frequency of IPV among a socially disadvantaged sample of urban ED patients. Confidential survey interviews were conducted with 1037 married/partnered study participants (46% male; 50% Hispanic; 29% African American) at a public safety-net hospital. Gender-stratified multilevel Tobit regression models were estimated for frequency of past-year physical IPV (perpetration and victimization) and frequency of severe IPV. Approximately 23% of participants reported IPV. Among men and women, impulsivity, adverse childhood experiences, substance use, and their spouse/partner’s hazardous drinking were associated with IPV frequency. Additionally, household food insufficiency, being fired or laid off from their job, perceived neighborhood disorder, and neighborhood demographic characteristics were associated with IPV frequency among women. Similar patterns were observed in models of severe IPV frequency. IPV prevention strategies implemented in urban ED settings should address the individual, household, and neighborhood risk factors that are linked with partner aggression among socially disadvantaged couples.

## 1. Introduction

In the U.S., prior research shows that urban emergency department (ED) patients have elevated rates of intimate partner violence (IPV) compared to those in the general household population [1,2,3,4]. For example, an analysis of Wave II data from the National Epidemiologic Survey of Alcohol-Related Conditions (NESARC), a nationally representative survey of U.S. adults, found that past-year IPV perpetration was reported by 4.0% of men and 6.9% of women. IPV victimization was reported by 5.6% of men and 5.0% of women [5]. In comparison, IPV prevalence based on urban ED studies is consistently higher. For example, a study of 712 non-urgent male ED patients found that 37% disclosed IPV involvement: 20% reported victimization only, 6% reported perpetration only, and 11% reported bidirectional/reciprocal IPV [3]. Houry et al. found that 28.2% of their ED-based sample reported IPV involvement; there were no gender differences in rates of victimization and perpetration [2]. Walton and colleagues reported 8.7% of their study’s ED sample disclosed IPV involvement. Among women, 6.0% reported IPV perpetration and 8.2% reported IPV victimization; among men, 2.3% reported IPV perpetration and 6.1% reported IPV victimization [4]. Bazargan-Hejazi et al. found that 16% of their ED sample reported some IPV involvement. Among this group, 31% reported perpetration only, 20% reported victimization only, and 49% reported bidirectional/reciprocal IPV, with no gender differences observed based on bivariate analysis [1].

The observed higher prevalence of IPV among patients who seek medical care at urban safety-net EDs can be partially explained by their social disadvantage and other characteristics that are associated with greater likelihood of IPV [6,7]. For example, compared to the general population, urban ED patients have elevated rates of substance use, mental health problems, and unemployment [8,9,10] and are more often exposed to aspects of the social environment that are linked with increased likelihood of IPV, such as neighborhood poverty [11]. Few ED-based IPV studies, however, have considered how neighborhood factors may be associated with IPV (e.g., [12]). Reviews of the literature on the association between neighborhood characteristics and IPV show that most of the research has been based on non-clinical samples obtained through county, city, state or national household surveys [13,14]. Although the results are not entirely consistent, aspects of neighborhood disadvantage (e.g., level of poverty or unemployment) are often linked to the occurrence of IPV [15,16,17,18]. Conceptually, research studies that examine the impact of neighborhood conditions on IPV are usually based on social disorganization theory [19]. This theory posits that socially disorganized neighborhoods can be characterized as having three components: low collective efficacy, weak informal local friendship networks, and low participation of residents in local organizations [19]. Aggregate neighborhood factors that inhibit community social organization include concentrated disadvantage, immigrant concentration, and residential instability. Weak or nonexistent social ties among residents of such neighborhoods may help create an environment where residents are unlikely to intervene in problem behaviors, such as public drunkenness or family violence. Under these conditions, higher rates of problem behaviors will be found in neighborhoods that lack the structure or resources to prevent or combat these problems when they arise [20]. Moreover, neighborhood exposure to social disadvantage can create stressors for one or both members of the couple in which IPV is more likely to occur [21].

Given the dearth of ED-based studies that have examined how neighborhood social disadvantage may be linked to IPV, the aim of this study is to analyze the extent that individual, household, and neighborhood-related factors are associated with frequency of IPV and severe IPV among a sample of married/partnered male and female urban ED patients. Previous analyses of the data found that 23% of study participants reported past-year physical IPV; among those reporting any IPV, most (57.3%) reported both victimization and perpetration [7]. In addition, among those who reported any IPV, approximately half reported severe IPV [6]. The current study extends these prior findings in several ways. First, rather than modeling IPV as a dichotomous outcome, the study’s outcome measures are frequency of IPV, and severe IPV. This is important because few ED-based studies (e.g., [22]) have quantified how frequently physically aggressive acts occur among those who report any IPV. Second, instead of statistically controlling for gender, a gender-stratified approach will be used to model study outcomes. This allows for a comparison of factors associated with IPV frequency among male and female study participants. Third, multilevel analysis will quantify the contribution of individual, household, and neighborhood characteristics to frequency of IPV, and frequency of severe IPV, among the sample.

This study is guided by the social-ecological conceptual framework developed through our IPV research among nationally representative samples [15,16,23,24], couples surveyed in community settings [25,26,27,28,29], and ecological analyses at the city [30] and state level [11]. The research demonstrates that an array of individual, couple, and environmental (neighborhood) factors are associated with the occurrence of IPV. At the individual level these factors include psychosocial characteristics, such as adverse childhood experiences and impulsivity, and alcohol-related behaviors [25,26,28]. Couple-level factors shown to be related to IPV are discrepant dyadic substance use patterns [31]. Neighborhood-related factors that are associated with IPV include perceived neighborhood disorder [23,24] and aspects of social disadvantage measured at the Census tract, Census block group, or zip code level, such as poverty [11,15,30]. For the current study, we hypothesize that individual factors (e.g., adverse childhood experiences; substance use) and household factors (e.g., household food insufficiency) will be positively associated with frequency of IPV, and severe IPV, and that neighborhood indicators of social disadvantage (e.g., Census tract poverty levels) will be positively associated with both outcomes. Understanding how these factors are related to IPV frequency among an urban ED sample is important because it can inform the development of IPV prevention and intervention strategies that can be implemented in socially disadvantaged communities [32].

## 2. Materials and Methods

### 2.1. Sample and Data Collection

This cross-sectional study was based among patients seeking non-emergency care at the ED of an urban Level I trauma center in Northern California. The hospital is part of a county-wide integrated public health care system. Study eligibility criteria were: 18–50 years old; English or Spanish speaker; resident of the county in which the hospital is located; and married, cohabiting, or in a romantic (dating) relationship for the past 12 months. Patients who were intoxicated, experiencing acute psychosis or suicidal or homicidal ideation, were cognitively/psychologically impaired and unable to provide informed consent, in custody by law enforcement, or in need of immediate medical attention were ineligible and excluded. The project’s protocol for the protection of human subjects was approved by the Alameda Health System Institutional Review Board.

Survey interviews were conducted with 1037 participants (53% female) between February and December 2017. Data collection procedures have been described elsewhere [6]. Briefly, trained bilingual research assistants (RAs) identified potentially eligible participants through the ED’s electronic patient information system and conducted face-to-face screening in the ED waiting room or in a treatment cubicle. The RAs conducted survey interviews in the patient’s room without others present using computer assisted personal interview (CAPI) techniques. Screening and survey interviews were conducted in English or Spanish based on participant language preference. Sample recruitment occurred during weekdays from 9 a.m.–9 p.m. Average survey interview completion time was 37 min (*SD* = 20.7). Participants provided informed consent and received a $30 grocery store gift card for completing the survey. No adverse psychological events occurred during data collection, nor to our knowledge were there any adverse events following data collection. See supplemental Figure S1 for the recruitment sequence.

### 2.2. Measurements

Intimate partner violence: Frequencies of past-12 month physical IPV victimization and perpetration were measured with the physical assault subscale of the CTS2—Revised Conflict Tactics Scale [33]. We created two outcome variables: any IPV and any severe IPV. For the former, the frequency of each act of perpetration and victimization was valued using the midpoint of each category: never (0), once (1), twice (2), 3–5 times (4), 6–10 times (8), and more than 10 times (15), then summed. For the latter, we used the same methodology to sum the frequency of only those items categorized as severe IPV per the CTS2: kicked; punched or hit with something that could hurt; beat up; choked; burned or scalded on purpose; slammed against a wall; used a knife or gun. Cronbach’s α for the physical assault subscale in the dataset under analysis was 0.88; when restricted to the perpetration or victimization items, α was 0.85 and 0.87, respectively.

Gender: Self-reported gender was coded as male or female.

Race/ethnicity: Participants were asked to name the racial or ethnic group(s) that best describes them from the following list: Native American Indian or Alaska Native; Filipino; Asian (not including Filipino); Black, African American; Latino, Hispanic; Native Hawaiian or other Pacific Islander (not including Filipino); White, Caucasian; some other race. Filipino was listed as a separate group from Asian due to many distinct Filipino cultural characteristics that derive from the Philippine’s history of Spanish colonization. Those who selected more than one category were categorized as multiracial/multiethnic. These groups were recoded into 5 racial/ethnic categories: white/Caucasian; Black/African American; multiracial/multiethnic; other; and Hispanic.

Age: This was used as a continuous variable.

Household food insufficiency: Participants rated their level of agreement with the statement, “In the past 12 months, the food we bought ran out and we didn’t have money to get more” (never; sometimes true; often true): We dichotomized and compared those who responded “sometimes” or “often” to those who responded “never” [34].

Perceived neighborhood disorder: This was measured with Hill and Angel’s 10-item measure of neighborhood disorder [35]. Items cover the extent to which assaults, muggings, drug dealing, gangs, unsafe streets, thefts, teenage pregnancy, abandoned houses, police not available, unsupervised children, and high unemployment, are neighborhood problems. Participants could select one of the following 3 categories to answer each item: Not a problem, somewhat of a problem, a big problem. Cronbach’s alpha was 0.88.

Fired/laid off from job: Participants were asked if in the past 12 months they were fired or laid off from a job. We dichotomized and compared those who responded “yes” to those who responded “no.”

Adverse childhood experiences (ACE): The modified brief ACE scale measures exposure to six adverse childhood experiences: (1) exposure to a mentally ill person in the home; (2) parent/caregiver alcoholism; (3) sexual abuse; (4) physical abuse; (5) psychological abuse; and (6) violence directed against the respondent’s mother [36]. The scale has been used in previous IPV studies [26,28]. The six exposures were summed to create the ACE variable (range = 0–6). Cronbach’s α was 0.74.

Impulsivity: This construct was measured with a 3-item scale used in prior IPV studies [25,27,37]. Response options ranged from 1 (quite a lot) to 4 (not at all). Items were reverse-coded and summed to create a score. Cronbach’s α was 0.79.

Post-traumatic stress disorder (PTSD): This 4-item measure is from the Primary Care Screener for PTSD [38]. A score of 3 or more is considered positive. Cronbach’s α was 0.83.

Frequency of intoxication. Participants were asked how often they had any kind of alcoholic beverage in the last 12 months. Those who consumed 1 or more drinks were asked additional questions about their alcohol consumption. To measure frequency of intoxication, they were asked, “During the past 12 months, about how many times did you drink enough to feel intoxicated or drunk, that is, when your speech was slurred, you felt unsteady on your feet, or you had blurred vision?” This question has been used in previous studies of IPV and drinking [39]. A recoded ‘frequency of intoxication’ variable was created in which abstainers were coded as ‘0,’ and the intoxication frequency values for all participants were log transformed due to skewed distribution.

Cannabis use: Participants were asked, “How many times during the past 12 months, or 365 days, did you use marijuana or hashish (weed, pot, hash) without a doctor’s instruction?” Days of marijuana use (0–365) were recorded. A new ‘days of cannabis use’ variable was created in which non-users were coded as ‘0,’ and the days of cannabis use values for all participants were log transformed due to skewness.

Spouse/partner hazardous drinking: Participants were asked to assess their spouse/partner’s drinking via the 3-item AUDIT-C (Alcohol Use Disorders Identification Test-Consumption) [40,41]. Male/female partners with a score above 4 or 3, respectively, were considered hazardous drinkers. Cronbach’s α was 0.81.

Spouse/partner cannabis use: Participants were asked, “During the past 12 months, or 365 days, did your spouse or partner use marijuana or hashish (weed, pot, hash) without a doctor’s instruction (yes/no)?” A dichotomous variable was created, coded ‘1’ for those who reported any past-year cannabis use and ‘0′ for those with no past-year use.

Neighborhood sociodemographic characteristics: Neighborhood-level sociodemographic characteristics used in previous IPV studies were selected for inclusion [11,27,30]. These are the percentage of residents within each respondent’s Census tract who reported being Black/African American, percentage reporting being white non-Hispanic/Latino, percentage of families in poverty (i.e., lower than 150% of the federal poverty line), median household income (per $10,000), and population density (thousands per square mile). These measures were obtained from GeoLytics Estimates Premium (GeoLytics, East Brunswick, NJ, USA).

### 2.3. Statistical Analysis

Data were analyzed using multilevel censored Tobit regression models allowing for neighborhood sociodemographic covariates measured at the Census tract level. Outcome counts were the number of past-year IPV events. Gender-specific models were estimated for frequency of any IPV, and for frequency of any severe IPV. The models include sociodemographic, psychosocial, substance use, and household factors previously shown to be associated with IPV [6,7,28]. Among the 1037 survey participants, cases in which the participant described themselves as transgender (*n* = 3) were dropped from the analysis due to small numbers. Data on 1 or more variables was missing from 60 male participants and 54 female participants; these cases were dropped from the analysis through listwise deletion. The analyses are therefore based on complete data from 424 males and 496 females and were conducted with Stata: Software for Statistics and Data Science 15.0. Missing data analysis showed that characteristics of dropped cases did not differ from cases with complete data, with the following exceptions: among males, dropped cases were less likely to be Hispanic (*t* = −2.29; *p* = 0.022), and more likely to be classified as ‘other’ race/ethnicity (*t* = 6.55; *p* < 0.001). Among females, dropped cases were less likely to be Black/African American (*t* = −3.65; *p* < 0.001), more likely to be classified as ‘other’ race/ethnicity (*t* = 3.54; *p* < 0.001), and less likely to have their spouse/partner classified as a hazardous drinker (*t* = −2.07; *p* = 0.039).

## 3. Results

### 3.1. Sample Characteristics

Sample characteristics are shown in Table 1. Average age was 34.044 years for females and 36.566 years for males. Most females self-reported their race/ethnicity as Hispanic (46.8%) or Black/African American (33.7%), as did most males (55.0% Hispanic; 27.1% Black/African American). Household food insufficiency was reported by 54.4% of females and 44.3% of males. Approximately 28% of females and 23% of males screened positively for PTSD. Among females, 14.3% had been fired or laid off from their job in the past year; among males, this was 17.9%. A larger proportion of females than males reported that their spouse/partner had used cannabis (27.2% vs. 18.6%). The spouse/partners of approximately 24% of females and 20% of males were categorized as hazardous drinkers based on the AUDIT-C screening criteria. Females and males reported 50.147 and 59.809 average days of cannabis use, respectively, in the past 12 months. Average past-year frequency of intoxication was 7.591 for females and 10.705 for males. Among those who had at least 1 IPV incident, the average IPV frequency was 17.198 among females and 9.602 among males. Moreover, average IPV victimization and perpetration frequencies were greater among females than males, as were average severe IPV frequency. Gender-specific correlation matrices are available in Supplementary Table S1.

### 3.2. Correlates of IPV Frequencies among Women

Results of the Tobit regression models among women are shown in Table 2. Participants who reported their race/ethnicity as Black/African American had greater frequency of any IPV (b = 23.050; *p* < 0.01) compared to those who identified as Hispanic. In terms of psychosocial characteristics, impulsivity (b = 2.425; *p* < 0.01) and adverse childhood experiences (b = 3.489; *p* < 0.05) were positively associated with IPV frequency. Those who reported being laid off or fired from their job reported greater IPV frequency (b = 14.817; *p* < 0.01) compared to those who did not report being laid off or fired. Days of cannabis use (b = 2.783; *p* < 0.01) was positively associated with IPV frequency. Regarding household factors, women who reported household food insufficiency had greater IPV frequency (b = 15.763; *p* < 0.01) compared to those who did not report food insufficiency. Those whose spouse/partner was categorized as a hazardous drinker had more IPV frequency compared to those whose spouse/partner wasn’t categorized as a hazardous drinker (b = 13.410; *p* < 0.01). In terms of neighborhood-related factors, perceived neighborhood disorder was positively associated with IPV frequency (b = 0.872; *p* < 0.05), as was Census tract median household (b = 3.968; *p* < 0.01). All of these factors were also significantly associated with frequency of severe IPV. In addition, percent of families in poverty at the Census tract level was related to this outcome (b = 0.414; *p* < 0.05).

### 3.3. Correlates of IPV Frequencies among Men

Results of the Tobit regression models among men are shown in Table 3. Participants whose race/ethnicity was categorized as “other” had greater frequency of any IPV (b = 11.042; *p* < 0.05) compared to those who identified as Hispanic. Regarding psychosocial factors, impulsivity (b = 1.279; *p* < 0.01) and adverse childhood experiences (b = 1.740; p < 0.05) were positively associated with IPV frequency. Frequency of intoxication (b = 1.800; *p* < 0.05) was positively associated with IPV frequency. In terms of household factors, those whose spouse/partner was categorized as a hazardous drinker had higher IPV frequency compared to those whose spouse/partner wasn’t categorized as a hazardous drinker (b = 11.803; *p* < 0.01). Except for frequency of intoxication, all of these factors were also associated with frequency of severe IPV. None of the neighborhood-related factors were associated with men’s IPV frequency or severe IPV frequency.

## 4. Discussion

The purpose of this study was to identify individual, household, and neighborhood factors associated with frequency of IPV, and severe IPV, among an urban ED sample of married/partnered men and women. In terms of IPV frequency, the results showed that among those who had reported any past-year physical IPV, women reported about twice as many IPV incidents as men (17.198 vs. 9.602). Comparability with the results of other studies is hampered by methodological differences. For example, Rhodes et al. [22] reported on mean IPV frequency among IPV-involved women who participated in a randomized clinical trial to reduce IPV and problem drinking at an urban ED. Their study used a brief version of the CTS2 (i.e., the CTS2S [42]), and their sample did not include men. Similarly, Hines and colleagues [43] analyzed physical IPV frequencies among a population-based sample using the CTS2. Their study, however, did not include women. The greater mean IPV frequencies reported by women compared to men in the current study is consistent with results from ED-based studies that show either no gender differences or greater IPV prevalence among women [1,2,3,4].

Our hypothesis that individual and household factors would be related to frequency of IPV, and severe IPV, was confirmed with some notable gender differences and similarities. For example, compared to Hispanic study participants, Black/African American women, and men in the “other” racial/ethnic category, had greater IPV frequency and severe IPV frequency. Previous ED-based studies have found that Blacks/African Americans are at elevated odds for IPV compared to other racial/ethnic groups [4,44]. Moreover, racial/ethnic disparities in IPV prevalence have been identified in the NESARC data for Native Americans, those who identify as multiracial, and Blacks/African Americans [45]. In the current study, men in the “other” racial/ethnic category comprise disparate racial identities that did not permit separate categorization in the analysis due to small numbers. The findings suggest that further research is needed to elucidate the factors that underlie these racial/ethnic differences in IPV frequency. Historical trauma, structural violence, and inequalities likely contribute to IPV disparities among Blacks/African Americans [46] and Native Americans [47].

Regarding individual psychosocial factors, adverse childhood experiences and impulsivity were positively associated with frequency of IPV, and severe IPV, among men and women. These findings are consistent with the results of other ED-based studies [1,48,49]. These factors have also been associated with IPV in studies of couples sampled in community settings [26,28]. Moreover, a large body of research shows that those exposed to ACEs are more likely to report mental, physical, and behavioral health problems, such as IPV, in adulthood [50,51,52]. The prevention of adverse childhood experiences should be a public health priority, especially among vulnerable populations, to reduce harmful exposures and their aftermath, including the propensity towards impulsive and aggressive behaviors. To achieve a population level impact on ACEs, the underlying social determinants of health will need to be addressed, such as socioeconomic adversity, unemployment, and poor or unsafe housing. Evidence-based parenting and home visitation programs are also important [53].

Being fired or laid off from a job was associated with frequency of IPV, and severe IPV, among women but not among men. Being fired or laid off constitutes a stressful life event that could heighten feelings of frustration and anger; in turn, conflict encountered while in these emotional states may be more likely to result in IPV. In a study of blue-collar couples in which the male partner was employed in the construction trade, couples in which the male partner was currently unemployed had greater odds of male-to-female partner violence (OR = 1.59, 95% CI 1.02, 2.48; *p* < 0.05) compared to couples in which the male partner was currently employed. In addition, the male partners’ number of months of unemployment were positively related to increased odds of female-to-male partner violence (OR = 1.11, 95% CI 1.02, 1.20; *p* < 0.05) [25]. The absence of an association between being fired/laid off and frequency of IPV among men in the current study is therefore somewhat unexpected.

As hypothesized, substance use-related factors were associated with frequency of IPV and severe IPV among the sample’s men and women. For example, men’s frequency of intoxication was positively related to frequency of IPV (but not severe IPV). Indicators of heavy drinking have been linked with IPV in previous ED-based studies [1,3,4,49] and in a wide variety of study settings and populations [54]. Results from the international GENACIS study show that self-reported IPV severity was greater for incidents in which one or both partners had been drinking. These findings were consistent for men and women and across respondents from 13 countries [55]. Proximal effects models, such as alcohol myopia, propose that the direct psychopharmacological effects of alcohol on cognitive functioning may result in increased aggression [56]. Spouse/partner hazardous drinking was also associated with frequency of IPV, and severe IPV, among male and female study participants. This finding underscores the importance of considering dyadic patterns of substance use in ED populations. From a prevention standpoint, reducing intoxication and hazardous drinking in couples may help to lessen IPV frequency. This is because these drinking behaviors can potentiate aggression among couples and can also serve as a source of conflict that can result in aggressive or violent behaviors.

The results showed that women’s cannabis use was positively related to frequency of IPV and severe IPV. Compared with the alcohol-IPV association, research evidence linking cannabis use with IPV has been less consistent [57]. For example, among a sample of men arrested for domestic violence, Shorey and colleagues found that cannabis use was significantly associated with IPV even after controlling for alcohol use and problems [58]. Yet among a sample of those arrested for domestic violence, Stuart et al. [59] found that women were less likely to perpetrate IPV on days in which cannabis was used [59]. Testa and colleagues found that among a sample of cannabis-using young couples, cannabis use episodes contributed to the occurrence of relationship conflict and verbal aggression but did not increase the likelihood of physical IPV [60]. As more U.S. states move towards the legalization of recreational cannabis use, it becomes increasingly important to consider how cannabis may contribute to the occurrence of IPV among ED populations, both directly and in concert with alcohol.

In terms of household factors, food insufficiency was positively associated with both outcomes among women but not men. While the causal mechanism is unknown, it is plausible that the stress associated with not having enough economic resources to ensure household food sufficiency could result in increased couple conflict, and thereafter IPV. What is unclear is why the association is only significant for women in the sample. From a prevention standpoint, the associations seen here and in other studies linking food insufficiency with IPV suggest that it may be vital to ensure that food-insecure households have adequate access to affordable, nutritious food [61,62,63]. In many U.S. urban centers, wealth inequity and housing displacement have been dramatically increasing; food insufficiency may mark the impact of these ecological trends on the health of the ED patient population [64].

Our hypothesis that neighborhood indicators of social disadvantage would be related to frequency of IPV, and severe IPV, was not confirmed for men, but was partially confirmed among women, for whom perceived neighborhood disorder was positively related to frequency of IPV. Gender differences regarding neighborhood factors have been observed in previous IPV studies. For example, in an analysis of a national sample of married/partnered men and women, Cunradi [23] found that perceived neighborhood disorder was associated with bidirectional IPV among men (OR = 1.61, 95% CI 1.39, 1.87); among women, neighborhood disorder moderated the association between drinking level and bidirectional IPV such that risk for IPV increased under conditions of high neighborhood disorder, and decreased to insignificant risk under conditions of low neighborhood disorder [23]. In a study of IPV perpetration and victimization among a national sample of Hispanic men and women, Cunradi [24] found that perceived neighborhood disorder was associated with increased odds of IPV perpetration among men (OR = 1.55, 95% CI 1.16, 2.09; *p* < 0.01), and with IPV victimization among men (OR = 1.36, 95% CI 1.04, 1.79; *p* < 0.05) and women (OR = 1.34, 95% CI 1.08, 1.67; *p* < 0.01).

At the Census-tract level, the results showed that neighborhood indicators of social disadvantage were not associated with IPV frequency among men. Among women, percent of families below 150% of poverty level was positively associated with frequency of severe IPV, and median household income was positively associated with frequency of IPV and severe IPV. In separate ecological studies, Cunradi and colleagues reported that percent of household below 150% of poverty level was positively related to IPV-related police calls in Sacramento, California [30], and to IPV-related ED visits throughout California over a 3-year period [11]. Census tract neighborhood poverty was related to IPV among a representative sample of U.S. couples, although the associations varied by couple race/ethnicity [15]. Yet in a multilevel analysis of a geographic sample of California couples, no association was found between IPV and neighborhood-related factors measured at the Census tract level [27]. In terms of the positive association between median household income and IPV, it may be that the relationship between income and IPV isn’t linear. It is likely that these two measures capture different things, with poverty being determined by the lower tail of the income distribution and median household income representing the middle. In order to address the impact of neighborhood factors on IPV, policies, programs and strategies will need to be developed that promote community economic development aimed at revitalizing the economic, physical, and social infrastructures of socially disadvantaged neighborhoods.

The study results need to be considered in the context of several limitations. First, the cross-sectional study design precludes causal inference. Second, the sample was obtained from a single urban ED, which may limit generalizability. Third, participants’ spouse/partners were not interviewed, and a lack of concurrent dyadic reports on the occurrence of IPV may result in its underestimation [65]. Moreover, participants may have overestimated or underestimated the substance use behaviors of their spouse/partner. Fourth, recall bias may have affected participants’ estimation of events or behaviors over the previous 12 months. Fifth, due to survey time constraints, no data were collected on psychological abuse, injury, or sexual coercion among participants and their spouse/partners. Lastly, Census tracts served as a proxy for neighborhoods. Because these units of measurement are designed for administrative Census purposes, they may not necessarily align with residents’ conceptions of what constitutes their neighborhood [66].

In terms of future directions, longitudinal study designs will be needed to assess temporal ordering of factors related to IPV. For example, studies that use intensive longitudinal data methods (e.g., ecological momentary assessment) can more precisely measure how behaviors (e.g., substance use and IPV) are related to each other over time. Because IPV is a dyadic behavior, future studies should strive to obtain data from both members of the couple [65]. Neighborhood-related factors should be incorporated into future IPV studies among socially disadvantaged populations.

## 5. Conclusions

Because urban EDs provide access to underserved populations, they offer an opportunity to investigate IPV-related health disparities. The current study is among the first to use a social-ecological framework to assess the relationships between individual, household and neighborhood factors and frequency of IPV and severe IPV among an urban ED sample. Based on a gender-stratified multilevel analysis, the findings showed that among the nearly one-quarter of the sample that experienced any past-year IPV, numerous incidents of past-year aggression were reported by men and women. Due to the cross-sectional study design, it is not possible to determine when acts of IPV victimization and perpetration occurred in relation to each other. However, the findings suggest that dyadic aggression may often occur in a bidirectional context among IPV-involved ED patients. Although there were some notable gender differences, the results provide evidence that an array of demographic, psychosocial, and substance use factors at the individual, household, and neighborhood levels are associated with frequency of IPV and should be considered when formulating IPV prevention and intervention strategies among socially disadvantaged ED populations.

## Figures and Tables

**Table 1:** Sample characteristics.

	Females (*n* = 496)	Males (*n* = 424)
	Mean (SD)	Mean (SD)
Age	34.044 (8.550)	36.566 (8.279)
Race/ethnicity:		
Hispanic	0.468 (0.499)	0.550 (0.498)
Black/African American	0.337 (0.473)	0.271 (0.445)
White/Caucasian	0.067 (0.249)	0.064 (0.244)
Multiracial/multiethnic	0.060 (0.239)	0.047 (0.212)
Other	0.069 (0.253)	0.068 (0.253)
Impulsivity	5.381 (2.585)	5.318 (2.512)
Adverse childhood experiences	1.486 (1.649)	1.144 (1.424)
PTSD	0.278 (0.449)	0.229 (0.421)
Perceived neighborhood disorder	6.861 (5.579)	5.941 (5.217)
Food insufficiency	0.544 (0.499)	0.443 (0.497)
Fired/laid off from job	0.143 (0.351)	0.179 (0.384)
Frequency of intoxication	7.591 (42.571)	10.705 (41.366)
Cannabis use (days)	50.147 (118.831)	59.809 (125.637)
Spouse/partner hazardous drinking	0.238 (0.426)	0.196 (0.397)
Spouse/partner cannabis use	0.272 (0.446)	0.186 (0.390)
IPV frequency (if >0)	17.198 (27.479)	9.602 (15.020)
IPV victimization frequency (if >0)	12.789 (21.520)	6.948 (10.417)
IPV perpetration frequency (if >0)	9.300 (27.053)	5.000 (7.669)
Severe IPV frequency (if >0)	9.273 (12.394)	6.590 (9.765)
Census tract measures:		
Population density (per 1000)	15.500 (7.338)	15.336 (7.628)
Percent Black/African American	27.027 (17.063)	24.779 (15.991)
Percent white/Caucasian	25.466 (13.145)	26.986 (12.449)
Median household income (per $10,000)	4.548 (1.978)	4.692 (1.841)
Percent families in poverty	19.837 (10.086)	18.224 (10.256)

**Table 2:** Correlates of IPV frequencies among women (*n* = 496 in 144 Census tracts).

Variable	Any IPV	Severe IPV
	b (SE)	b (SE)
Age	−0.211 (0.239)	−0.098 (0.159)
Race/ethnicity: (ref: Hispanic)		
Black/African American	23.050 ** (5.436)	10.783 ** (3.610)
White/Caucasian	3.158 (9.972)	3.651 (6.159)
Multiracial/multiethnic	14.183 (8.053)	5.869 (5.168)
Other	9.648 (9.293)	−4.701 (8.497)
Impulsivity	2.425 ** (0.774)	1.401 ** (0.488)
Adverse childhood experiences	3.282 * (1.290)	1.712 * (0.832)
PTSD	3.310 (4.671)	3.468 (2.903)
Perceived neighborhood disorder	0.872 * (0.402)	0.453 (0.261)
Food insufficiency	15.763 ** (4.719)	6.275 * (3.157)
Fired/laid off from job	14.817 ** (4.854)	6.882 * (3.021)
Frequency of intoxication	1.633 (1.703)	−0.389 (1.042)
Cannabis use (days)	2.783 ** (0.985)	1.384 * (0.614)
Partner hazardous drinking	13.410 ** (4.646)	9.837 ** (2.944)
Partner cannabis use	−5.006 (5.270)	0.500 (3.319)
Census tract measures:		
Population density (per 1000)	−0.025 (0.352)	−0.011 (0.229)
Percent Black/African American	−0.159 (0.163)	−0.088 (0.106)
Percent white/Caucasian	−0.203 (0.243)	−0.025 (0.156)
Median household income (per $10,000)	3.968 ** (1.452)	2.047 * (0.896)
Percent families in poverty	0.483 (0.290)	0.414 * (0.187)
Constant	−92.311 ** (18.304)	−61.991 ** (12.306)

* *p* < 0.05; ** *p* < 0.01.

**Table 3:** Correlates of IPV frequencies among men (*n* = 424 in 148 Census tracts).

Variable	Any IPV	Severe IPV
	b (SE)	b (SE)
Age	−0.188 (0.145)	0.038 (0.147)
Race/ethnicity: (ref: Hispanic)		
Black/African American	2.790 (3.316)	−2.033 (3.492)
White/Caucasian	4.871 (4.635)	−0.541 (4.726)
Multiracial/multiethnic	5.901 (5.042)	2.003 (4.969)
Other	11.042 * (4.459)	11.520 ** (4.122)
Impulsivity	1.279 ** (0.480)	1.304 ** (0.482)
Adverse childhood experiences	1.740 * (0.846)	1.762 * (0.853)
PTSD	5.169 (2.754)	1.857 (2.745)
Perceived neighborhood disorder	0.239 (0.238)	0.449 (0.246)
Food insufficiency	2.970 (2.399)	−1.343 (2.582)
Fired/laid off from job	3.245 (2.832)	0.242 (2.874)
Frequency of intoxication	1.800 * (0.825)	0.168 (0.843)
Cannabis use (days)	0.610 (0.612)	1.047 (0.615)
Partner hazardous drinking	11.803 ** (2.674)	7.365 ** (2.701)
Partner cannabis use	4.090 (3.272)	3.615 (3.307)
Census tract measures:		
Population density (per 1000)	−0.137 (0.196)	−0.130 (0.202)
Percent Black/African American	−0.196 (0.101)	−0.131 (0.104)
Percent white/Caucasian	−0.146 (0.141)	−0.177 (0.144)
Median household income (per $10,000)	−0.558 (1.038)	−0.124 (1.054)
Percent families in poverty	0.074 (0.192)	−0.075 (0.206)
Constant	−17.421 (11.901)	−25.699 * (12.428)

* *p* < 0.05; ** *p* < 0.01.

## Data Availability

The data are not publicly available due to Institutional Review Board restrictions.

## Supplementary Materials

The following are available online at https://www.mdpi.com/1660-4601/18/1/222/s1, Figure S1: Recruitment sequence, Table S1: Correlation matrices.
