# Supplementary material for: Frequency of Intimate Partner Violence among an Urban Emergency Department Sample: A Multilevel Analysis
Source: Int J Environ Res Public Health. 2020 Dec 30;18(1):222. doi: 10.3390/ijerph18010222 (PMC7796012; doi:10.3390/ijerph18010222)

Supplemental Table S1 – Correlation Matrices

A. Correlation matrix of outcome measure and respondent/Census-tract characteristics among females:

	IPV	AGE	HIS	BLA	WHI	MUL	OTH	IMP	ACE	PTS	PND	INT	CAN	FIH	UNE	HAZ	PMJ	DEN	PBL	PWH	MHH	POV
IPV	1																					
AGE	-0.046	1																				
HIS	-0.195*	0.077	1																			
BLA	0.195*	-0.123*	-0.668*	1																		
WHI	-0.017	-0.027	-0.250*	-0.190*	1																	
MUL	0.066	0.003	-0.238*	-0.181*	-0.068	1																
OTH	-0.026	0.102*	-0.254*	-0.193*	-0.072	-0.069	1															
IMP	0.268*	-0.077	-0.160*	0.148*	0.020	0.084	-0.059	1														
ACE	0.170*	0.010	-0.115*	-0.008	0.128*	0.151*	-0.027	0.185*	1													
PTS	0.183*	0.002	-0.113*	-0.004	0.087	0.220*	-0.062	0.257*	0.360*	1												
PND	0.133*	-0.073	0.018	0.045	-0.086	0.105*	-0.134*	0.119*	0.246*	0.223*	1											
INT	0.122*	-0.085	-0.261*	0.143*	0.202*	0.140*	-0.084	0.218*	0.156*	0.154*	0.077	1										
CAN	0.257*	-0.228*	-0.355*	0.332*	0.041	0.136*	-0.088*	0.228*	0.114*	0.127*	0.106*	0.321*	1									
FIN	0.188*	-0.019	-0.035	0.044	-0.064	0.079	-0.024	0.223*	0.172*	0.216*	0.302*	-0.040	0.163*	1								
UNE	0.196*	-0.076	-0.118*	0.111*	0.006	0.041	-0.0200	0.172*	0.079	0.106*	0.065	0.118*	0.182*	0.143*	1							
HAZ	0.205*	0.094*	-0.059	0.073	-0.016	0.037	-0.039	0.191*	0.082	0.139*	0.108*	0.309*	0.100*	0.083	0.015	1						
PMJ	0.208*	-0.186*	-0.419*	0.398*	0.109*	0.092*	-0.112*	0.201*	0.232*	0.186*	0.162*	0.303*	0.533*	0.159*	0.190*	0.201*	1					
DEN	0.040	-0.040	0.125*	-0.025	-0.098*	-0.049	-0.057	-0.013	0.069	0.024	0.080	-0.023	-0.016	0.027	-0.033	0.0661	-0.079	1				
PBL	0.060	0.029	-0.208*	0.237*	-0.009	0.084	-0.102*	0.050	0.067	0.143*	0.290*	0.062	0.145*	0.114*	0.08	0.110*	0.203*	-0.188*	1			
PWH	-0.044	-0.002	-0.039	-0.099*	0.246*	-0.077	0.094*	0.008	-0.044	-0.100*	-0.319*	0.030	-0.117*	-0.115*	-0.041	-0.119*	-0.077	-0.292*	-0.525*	1		
MHH	0.005	0.061	-0.069	-0.036	0.160*	-0.014	0.058	0.020	-0.071	-0.004	-0.283*	0.039	-0.076	-0.069	-0.027	-0.072	-0.005	-0.423*	-0.305*	0.665*	1	
POV	0.077	-0.071	0.061	0.051	-0.141*	-0.003	-0.074	0.006	0.005	0.045	0.309*	0.004	0.097*	0.061	0.054	0.072	0.043	0.334*	0.465*	-0.642*	-0.686*	1

Key: IPV=Intimate partner violence frequency; AGE=respondent age; HIS=Hispanic; BLA=Black/African American; WHI=White; MUL=multiracial/multiethnic; OTH=Other race/ethnicity; IMP=impulsivity; ACE=Adverse childhood events; PTS=Post Traumatic Stress Disorder; PND=perceived neighborhood disorder; INT=intoxication frequency; CAN=days of cannabis use; FIN=food insufficiency; UNE=fired or laid off; HAZ=spouse/partner hazardous drinking; PMJ=spouse/partner cannabis use; DEN=Census tract population density; PBL=Census tract percent Black; PWH=Census tract percent white; MHH=Census tract median household income; POV=Census tract percent families in poverty.

*indicates a significant correlation (p <= 0.05)

B. Correlation matrix of outcome measure and respondent/Census-tract characteristics among males:

	IPV	AGE	HIS	BLA	WHI	MUL	OTH	IMP	ACE	PTS	PND	INT	CAN	FIH	UNE	HAZ	PMJ	DEN	PBL	PWH	MHH	POV
IPV	1																					
AGE	-0.043	1																				
HIS	-0.131*	-0.076	1																			
BLA	0.011	0.118*	-0.674*	1																		
WHI	0.078	0.0172	-0.288*	-0.159*	1																	
MUL	0.037	-0.029	-0.246*	-0.136*	-0.058	1																
OTH	0.133*	-0.050	-0.299*	-0.165*	-0.071	-0.060	1															
IMP	0.256*	-0.106*	-0.159*	0.0473	0.117*	0.078	0.051	1														
ACE	0.202*	0.030	-0.082	-0.099*	0.225*	0.228*	-0.073	0.263*	1													
PTS	0.235*	-0.132*	-0.195*	0.047	0.203*	0.091	0.030	0.300*	0.308*	1												
PND	0.135*	-0.062	0.009	0.031	-0.053	0.071	-0.081	0.175*	0.200*	0.211*	1											
INT	0.293*	0.022	-0.107*	0.044	0.041	0.056	0.046	0.247*	0.163*	0.242*	0.145*	1										
CAN	0.155*	-0.132*	-0.437*	0.392*	0.146*	0.141*	-0.090	0.218*	0.163*	0.194*	0.103*	0.178*	1									
FIN	0.111*	-0.009	0.054	-0.053	0.001	0.025	-0.035	0.104*	0.147*	0.169*	0.212*	0.091	0.044	1								
UNE	0.175*	-0.042	-0.133*	0.102*	0.029	0.041	0.020	0.164*	0.104*	0.185*	0.070	0.125*	0.095	0.041	1							
HAZ	0.294*	-0.011	-0.151*	0.127*	0.066	0.030	-0.016	0.184*	0.134*	0.156*	0.107*	0.284*	0.192*	0.062	0.110*	1						
PMJ	0.240*	-0.163*	-0.322*	0.280*	0.099*	0.094	-0.034	0.287*	0.165*	0.215*	0.144*	0.229*	0.571*	0.073	0.155*	0.298*	1					
DEN	0.019	-0.004	0.075	-0.098*	-0.037	-0.016	0.074	-0.093	-0.071	-0.032	0.052	-0.026	-0.077	0.046	-0.073	-0.049	-0.077	1				
PBL	-0.021	-0.090	-0.298*	0.324*	-0.088	0.177*	-0.047	0.045	0.141*	0.128*	0.260*	0.095*	0.246*	0.000	0.043	0.111*	0.135*	-0.116*	1			
PWH	-0.038	0.053	-0.010	-0.042	0.159*	-0.069	-0.001	0.045	-0.047	-0.086	-0.326*	-0.058	0.0156	-0.102*	0.021	-0.055	0.034	-0.388*	-0.464*	1		
MHH	-0.023	0.096*	-0.001	-0.045	0.108*	-0.088	0.0504	0.036	0.013	-0.026	-0.257*	-0.008	-0.021	0.003	0.057	0.042	-0.021	-0.480*	-0.323*	0.566*	1	
POV	0.041	-0.112*	-0.049	0.047	-0.104*	0.093	0.035	-0.006	-0.006	0.054	0.282*	0.090	0.026	0.013	-0.082	-0.022	-0.010	0.404*	0.470*	-0.632*	-0.751*	1

Key: IPV=Intimate partner violence frequency; AGE=respondent age; HIS=Hispanic; BLA=Black/African American; WHI=White; MUL=multiracial/multiethnic; OTH=Other race/ethnicity; IMP=impulsivity; ACE=Adverse childhood events; PTS=Post Traumatic Stress Disorder; PND=perceived neighborhood disorder; INT=intoxication frequency; CAN=days of cannabis use; FIN=food insufficiency; UNE=fired or laid off; HAZ=spouse/partner hazardous drinking; PMJ=spouse/partner cannabis use; DEN=Census tract population density; PBL=Census tract percent Black; PWH=Census tract percent white; MHH=Census tract median household income; POV=Census tract percent families in poverty.

*indicates a significant correlation (p <= 0.05)

Figure 1. Study sample recruitment.

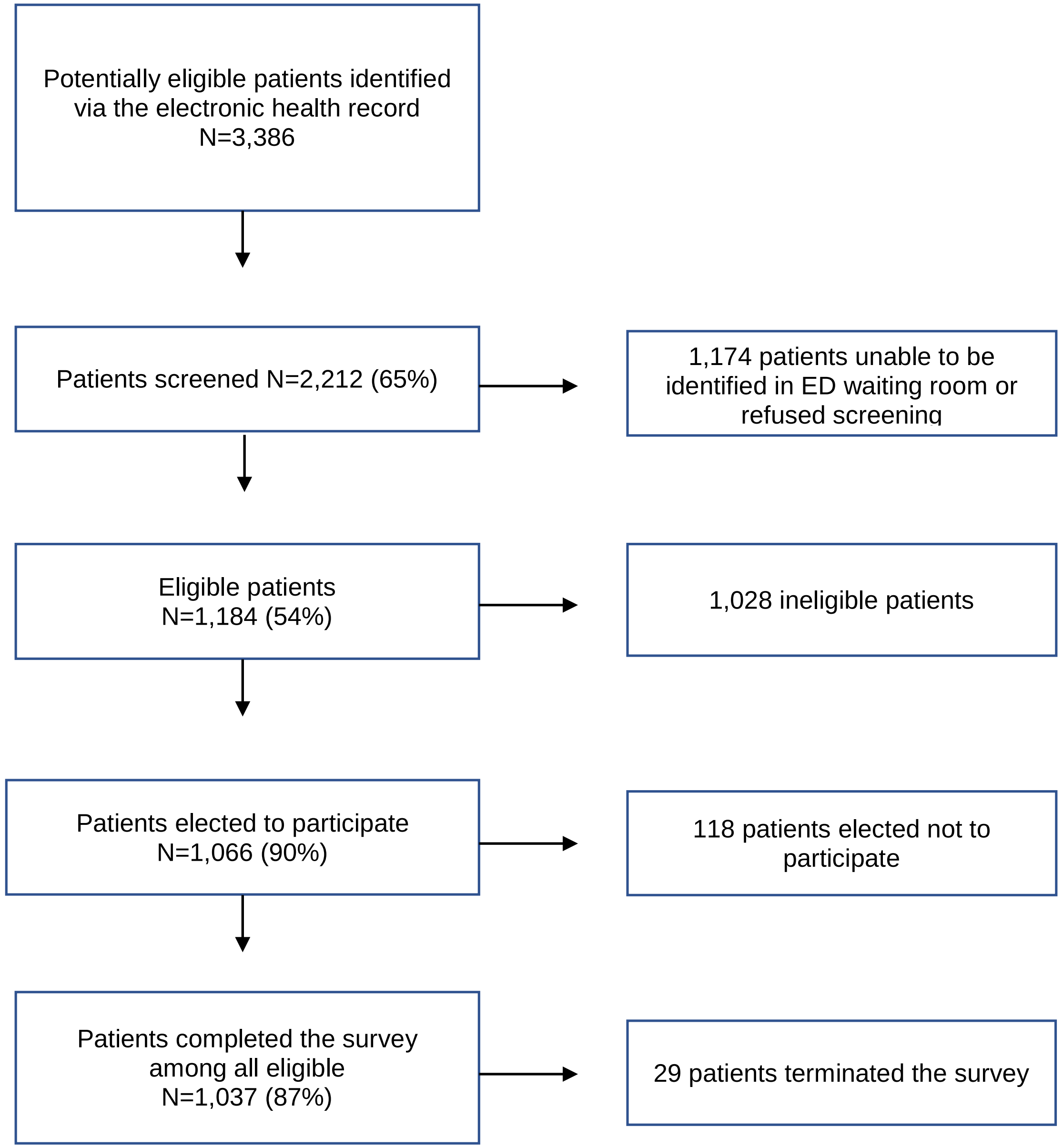

Supplement: Supplementary file 1 [file ijerph-18-00222-s001.pdf]
